# Effect of Ink and Pretreatment Conditions on Bioethanol and Biomethane Yields from Waste Banknote Paper

**DOI:** 10.3390/polym13020239

**Published:** 2021-01-12

**Authors:** Omid Yazdani Aghmashhadi, Lisandra Rocha-Meneses, Nemailla Bonturi, Kaja Orupõld, Ghasem Asadpour, Esmaeil Rasooly Garmaroody, Majid Zabihzadeh, Timo Kikas

**Affiliations:** 1 Department of Wood and Paper Engineering, Sari University of Agricultural Sciences and Natural Resources, Km 9 Farah Abad Road, Sari 66996-48181, Mazandaran Province, Iran; asadpur2002@yahoo.com (G.A.); m.zabihzadeh@sanru.ac.ir (M.Z.); 2 Institute of Technology, Chair of Biosystems Engineering, Estonian University of Life Sciences, Kreutzwaldi 56, 51006 Tartu, Estonia; Timo.Kikas@emu.ee; 3 Institute of Technology, University of Tartu, 50411 Tartu, Estonia; Nemailla.Bonturi@ut.ee; 4 Institute of Agricultural and Environmental Sciences, Estonian University of Life Sciences, Kreutzwaldi 5, 51006 Tartu, Estonia; Kaja.Orupold@emu.ee; 5 Department of Bio-refinery Engineering, Faculty of New Technologies Engineering, Shahid Beheshti University, Zirab P.O. Box 47815-168, Mazandaran, Iran; e_rasooly@sbu.ac.ir

**Keywords:** anaerobic digestion, biofuel, biomass, cotton-based waste, closed-loop, lignocellulose

## Abstract

Waste banknote paper is a residue from the banking industry that cannot be recycled due to the presence of ink, microbial load and special coating that provides protection against humidity. As a result, waste banknote paper ends up being burned or buried, which brings environmental impacts, mainly caused by the presence of heavy metals in its composition. To minimize the environmental impacts that come from the disposal of waste banknote paper, this study proposes to produce value-added products (bioethanol and biogas) from waste banknote paper. For this, the effect of ink and pretreatment conditions on bioethanol and biomethane yields were analyzed. Waste banknote paper provided by the Central Bank of Iran was used. The raw material with ink (WPB) and without ink (WPD) was pretreated using sulfuric acid at different concentrations (1%, 2%, 3%, and 4%) and the nitrogen explosive decompression (NED) at different temperatures (150 °C, 170 °C, 190 °C, and 200 °C). The results show that the use of NED pretreatment in WPD resulted in the highest glucose concentration of all studies (13 ± 0.19 g/L). The acid pretreatment for WPB showed a correlation with the acid concentration. The highest ethanol concentration was obtained from the fermentation using WPD pretreated with NED (6.36 ± 0.72 g/L). The maximum methane yields varied between 136 ± 5 mol/kg TS (2% acid WPB) and 294 ± 4 mol/kg TS (3% acid WPD). Our results show that the presence of ink reduces bioethanol and biogas yields and that the chemical-free NED pretreatment is more advantageous for bioethanol and biogas production than the acid pretreatment method. Waste banknote paper without ink is a suitable feedstock for sustainable biorefinery processes.

## 1. Introduction

The population growth and economic development have led to an increase in the production and consumption of materials and resources to satisfy global demand. However, this has also led to an increase in the amount of waste produced, such as municipal solid waste, industrial waste, and wastepaper [1,2]. Instead of being discarded after primary use, these residues can be used for the production of value-added products e.g., production of low-cost biofuels [3,4]. Among wastepaper, great attention has been paid to waste banknotes since they cannot be recycled due to their properties, such as the presence of pathogenic bacteria, heavy ink, and presence of formaldehyde melamine resin that provides protection against humidity and makes the digestion process more challenging [5,6,7,8].

The most common handling options for banknote paper are incineration or landfilling [9]. However, these strategies increase air pollution, and at the same time, pollute soils, and groundwater [10]. As a result, there is a search for more suitable and environmentally friendly handling options that can be applied to waste banknote paper. Banknote paper is to a large extent made of cotton and thus, has a high percentage of cellulose and low amounts of lignin in its composition, which makes it a suitable feedstock for the production of biofuels [7]. As banknote paper has high amounts of ink in its composition, a deinking process is required before its further treatment. The deinking process is composed of three sequential steps: pulping, soaking, and screening [7]. In the pulping stage, chemical and mechanical methods are used to separate ink and other non-fibrous contaminants from the fibrous material [7,11,12]. In the soaking stage, sodium hydroxide is used at high temperatures, followed by sieving and rinsing to separate and remove ink particles from the pulp [13]. Finally, in the screening process, a centrifugal cleaning is used to select and separate contaminants from the pulp fibers. Banknote paper has high amounts of alpha-cellulose in its composition (88 to 96%) which makes it a suitable feedstock for bioethanol and biogas production. However, banknote paper has also a very crystalline structure that requires effective and harsh pretreatments methods to make the cellulose accessible for enzymatic hydrolysis [7,14].

Bioethanol production is composed of four sequential steps: pretreatment, hydrolysis, fermentation, and distillation [15]. A wide variety of chemical, physical, biological, and physio-chemical pretreatment methods have been reported in the literature as effective in breaking down the plant cell wall and in making cellulose accessible to the enzymes [16]. Chemical pretreatment methods include acid, alkali, ionic liquids, and organosolv. Although chemical pretreatment methods have been widely used and are effective in dissolving lignin and hemicellulose, they are still expensive because of the high cost of chemicals [17]. Besides, several inhibitors, such as aliphatic carboxylic acids, phenolic compounds, and furans, are formed during chemical pretreatment, which may disrupt subsequent hydrolysis and fermentation processes [18,19]. Physical pretreatment methods include comminuting, irradiation, and freezing, while biological pretreatment methods use fungi, enzymes, or microorganisms to break down the lignin and hemicellulose bonds [14,16]. Combined physical and chemical pretreatment methods include ammonia fiber expansion, steam explosion (SE), carbon dioxide explosion (CO_2_ explosion, and NED). 

From all the pretreatment methods reported before, great attention has been paid to chemical-free pretreatment methods (e.g., SE, CO_2_ explosion and NED) since these methods reduce environmental impacts and costs that incur when chemicals are used [20]. Steam explosion pretreatment method uses high pressure, saturated steam, and a rapid decompression to disrupt the plant cell wall and dissolve the hemicellulose [16]. CO_2_ explosion uses supercritical CO_2_ and low pretreatment temperatures to improve the digestibility of the biomass [14]. NED is one of the most effective physical and chemical pretreatments methods reported in the literature [21]. This pretreatment method used nitrogen and high temperatures to open the biomass structure more effectively. NED is economically and environmentally attractive because neither catalysts nor chemicals are used in the processes [20]. However, the principle of operation and the processes that take place during this pretreatment still need further research [19].

Waste paper, and particularly waste banknote paper can also be used as a raw material for biogas production since it is a cheap source of organic material [22]. Anaerobic digestion is a biological process that occurs in the absence of oxygen and uses micro-organisms to convert organic waste into high-quality nutrient-rich fertilizer and yield energy in the form of biogas [23]. Biogas is mainly composed of methane (50–70%) and carbon dioxide (30–50%). Minor compounds include vapor water, nitrogen, oxygen, hydrogen sulfide, and ammonia [24]. Biogas can be upgraded to produce biomethane. 

This study aims to investigate the potential of waste banknote paper (with and without ink) for bioethanol and biomethane production. For this purpose, two different pretreatment methods were applied—nitrogen explosive decompression and sulfuric acid pretreatment methods. 

## 2. Materials and Methods 

**Figure 1:**
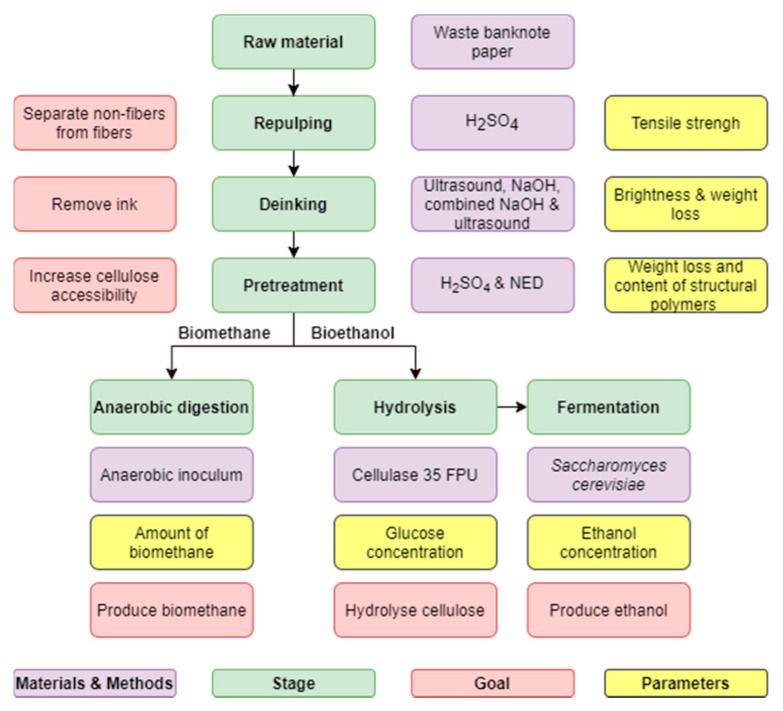
Production pathway utilized in this study to evaluate the potential of waste banknote paper for bioethanol and biomethane production.

The production pathway utilized in this study to evaluate the potential of waste banknote paper for bioethanol and biogas production is illustrated in Figure 1.

### 2.1. Raw Material

Waste banknote paper obtained from the Central Bank of the Islamic Republic of Iran was used as a raw material in this study. After collection, the waste banknote paper was placed in the laboratory for 48 h to ensure equilibrium moisture content. After measuring the moisture content, the raw material was stored in bags until use. 

### 2.2. Repulping 

Wet strength is one of the main factors that should be taken into account when talking about waste banknote paper. This property of the paper is related to the presence of melamine formaldehyde, which is added to the banknote paper to increase its mechanical resistance and reduce water absorption. Therefore, the repulping process is required to break down melamine resin cross-links. In order to find the best conditions for the repulping process, the tensile strength was measured at different pHs (from pH = 1 to pH = 12). The lowest tensile strength indicates the best repulping conditions. The tensile strength was measured with a tensile tester, from FRANK-PTI GMBH company (Birkenau, Germany), following the standard method TAPPI T456 OM-15 [25]. For repulping, 0.5 mL of 98% sulfuric acid was added to 30 g of pulp, and distilled water was added to a 500 mL Erlenmeyer flask until a total working volume of 300 mL was reached. The samples were placed on a hot plate with a magnetic stirrer (M TOPS MS300HS, from Misung Scientific Co., Ltd., Gyeonggi-do, Korea) at 90 °C to 95 °C, and stirred at 500 rpm, for 90 min [26,27]. All the experiments were performed in triplicate.

### 2.3. Deinking

After repulping, the samples were deinked using three deinking methods: chemical (1%, 2%, 3%, and 4% sodium hydroxide), ultrasonic (30Am, for 5 min) and integrated ultrasonic and sodium hydroxide. In the ultrasonic deinking method, a Q700 Sonicator was used (from Qsonica, Newtown, USA). In the deinking process, 3g (dry weight) of pulp was put in 100 mL Erlenmeyer flasks and treated at a temperature of 90–95 °C. To increase the contact during the process, a mixer model FTDS 41 from Sci Finetech Co (Seoul, Korea) was used for 90 min, at 500 rpm. In order to separate and remove the ink from the fibrous materials, the samples were washed using distilled water and a 200 mesh filter. After finishing the deinking process, a new paper was produced, for the weight loss, tensile strength, and brightness measurements. The brightness properties of the papers were measured according to the standard T452 om 08 using a Zb-a Powders brightness colorimeter testing from Hangzhou Zhibang Automation Technology Co. Ltd. (Hangzhou, China) [7,26]. The sodium hydroxide with a purity of 100% was from Merck Group (Darmstadt, Germany).

### 2.4. Pretreatment

#### 2.4.1. Chemical Pretreatment

H_2_SO_4_ with 98% purity was used for the chemical pretreatment of the banknote pulp samples. The acid was added in different concentrations (1%, 2%, 3%, and 4%) to Erlenmeyer flasks with 100g (dry weight) deinked waste banknote pulp (WPD) and non-de-inked waste banknote pulp (WPB). The samples were autoclaved for 30 min at 121 °C. After the pretreatment, solid and liquid fractions of the samples were separated by centrifugation (Thermo Scientific Heraeus megacentrifuge, Waltham, USA) at 10,000 rpm for 30 min. Samples from the solid fraction were left to dry at the atmospheric pressure and their weight loss was calculated.

#### 2.4.2. Physio-Chemical Pretreatment

In NED pretreatment, 800 mL of distilled water was added to 100 g of dried WPD and WPB. The samples were mixed and pretreated at 150 °C, 170 °C, 190 °C, and 200 °C, at a pressure of 30 bars using compressed nitrogen gas. After reaching the desired temperatures, the samples were cooled down to 80 °C and the pressure was released from the vessel in an explosive manner [28]. After the pretreatment, solid and liquid fractions of the samples were separated by centrifugation (Thermo Scientific Heraeus megacentrifuge, Waltham, USA) at 10,000 rpm for 30 min.

### 2.5. Enzymatic Hydrolysis 

Pretreated WPD and WPB samples from the solid fraction were used to make a broth with 2.5% dry matter content that was further used for the enzymatic hydrolysis. The enzyme complex Accellerase 1500 (from DuPont de Nemours) was used at a ratio of 0.3 mL per g of biomass. Distilled water was added to the flasks to obtain a total working volume of 200 mL. The hydrolysis took place for 72 h, at a temperature of 50 °C, under constant stirring in the orbital shaker (IKA^®^-Werke GmbH & Co. KG, Staufen im Breisgau, Germany) [18]. After the hydrolysis, the solid and liquid fractions of the samples were separated by centrifugation (Thermo Scientific Heraeus megacentrifuge, Waltham, USA) at 10,000 rpm for 30 min. All the experiments were performed in triplicate.

### 2.6. Fermentation

Liquid samples that were obtained from the hydrolysis stage were further fermented using the yeast *Saccharomyces cerevisiae.* This yeast was added to the samples from the liquid fraction at the ratio of 0.025 g/g. The fermentation process lasted 7 days and it was carried out at 25 °C [20].

### 2.7. Biomethane Potential

The biomethane potential (BMP) test used in this article is based on an adapted version of the protocol reported by Angelidaki et al. [29]. The inoculum was obtained from Tartu municipal wastewater treatment plant (Estonia), sieved through 2 mm mesh and pre-incubated for 4 days at 36 °C for degasification, before use [24]. The experiments were carried out in 575 mL plasma bottles with a working volume of 300 mL using the substrate to inoculum volatile solids (VS) ratio of 0.25. The blank test (just inoculum without substrate) was included to study the biogas and methane production of the inoculum, which later was subtracted from that of the samples with the substrate. The test bottles were flushed with N_2_ to assure anaerobic conditions. The BMP tests were performed at 36 °C in the lab incubator (Memmert GmbH + Co. KG, Schwabach, Germany) for 32 days (up until the methane content was constant). The experiments were carried out in triplicate. During BMP test the pressure in the headspace of test bottles was measured with pressure meter BMP-Testsystem WAL (WAL Mess- und Regelsysteme GmbH). 

### 2.8. Chemical Analysis

The neutral detergent fiber (NDF), acid detergent fiber (ADF) and acid detergent lignin (ADL) were determined using an ANKOM 2000 I, fiber Analyzer (ANKOM Technology Corporation, NY 14502, USA). 

The dry matter content (TS) was analyzed with a moisture analyzer Ohaus MB 45. The volatile solids (VS) were analyzed as loss on ignition at 550 °C. The pH of the samples before and after pretreatment, hydrolysis, and fermentation was measured using a pH meter, model SevenCompact pH/Ion S220 from Mettler-Toledo AG (Schwerzenbach, Switzerland).

Glucose, glycerol, acetic acid, and ethanol were quantified by HPLC (LC-2030C Plus, Shimadzu, Kyoto, Japan) equipped with a refractive index detector (RID-20A, Shimadzu, Kyoto, Japan) using a Rezex ROA Organic Acid column (Phenomenex, Torrance, CA, USA) column at 45 °C, and isocratic elution at 0.6 mL/min of 5 mmol/L H_2_SO_4_.

The quantification of methane in the produced biogas was done by gas chromatography (CP-4900 Micro-GC, from Varian Inc., Palo Alto, USA).

### 2.9. Calculations

The hydrolysis and fermentation efficiency were calculated based on Equation 1 and Equation (2), respectively [30].

(1) $$E_{\mathrm{HY}}\;=\;\frac{m_{\mathrm{glc}}}{m_{\mathrm{cel}}.1.11}.\;100\%$$

(2) $$E_{F}\;=\;\frac{C_{\mathrm{eth}}}{C_{\mathrm{glc}}.0.51}.\;100\%$$

where E_HY_ is the hydrolysis efficiency, m_glc_ the amount of glucose, m_cel_, the amount of cellulose, 1.11 is the conversion factor of cellulose to glucose, E_F_ is the fermentation efficiency, C_eth_ is the concentration of ethanol, c_glc_ is the concentration of glucose, and 0.51 is the conversion factor of glucose to ethanol 

The methane production was fitted and modelled in the software Graph-Pad Prism 5.0 with a non-linear regression model, using the equation Gompertz growth (Equation (3)).

(3) $${Y\;=\;Y}_{M}*{\left(\frac{Y_{0}}{Y_{M}}\right)}^{\exp\left(-K*X\right)}$$

where Y is the cumulative methane produced (L/kg TS), Y_M_ is the maximum population (L/kg TS), Y_0_ is the starting population (L/kg TS), K is the lag time (d^−1^), X is time (days).

### 2.10. Statistical Analysis

The statistical analysis was performed in software Graph-Pad Prism 5.0. The normal distribution of the results was investigated using the Shapiro-Wilk normality test. For glucose and ethanol yields, the differences between the variables were studied using two-way ANOVA, followed by the post hoc test Tukey’s multiple comparisons test. For biomethane yields, one-way ANOVA was used to investigate the statistically significant differences between the means of the different variables. The *post hoc* Dunn’s multiple comparisons test. The results were statistically significant when *p* ≤ 0.05 (confidence interval 95%).

## 3. Results

The results of the deinking and repulping process are reported in a previous paper published by the authors [26].

### 3.1. Chemical Composition

The results of the chemical composition, ash content, moisture, pH, and weight loss of WPB and WPD after being pretreated with NED and H_2_SO_4_ are reported in Table 1. The percentage of cellulose, hemicellulose, lignin, and ash for untreated WPB and WPD was 77–89%, 3–7%, 1–2%, and 0.6–1%, respectively. The cellulose content of WPB varied between 78% and 85%, while for WPD samples it varied between 84% and 89%. The percentage of hemicellulose differed from 1% and 6% for WPB samples, and from 1% to 8% for WPD samples. The amount of lignin and ash in all the samples was less than 2% and 1%, respectively. The moisture content in all the samples varied between 3% and 9%, and the pH between 5 and 6. The weight loss of all the samples was ≤ 10.2%. Statistically significant differences were found in the cellulose content of samples that were pretreated with acid 3% WPD and samples that were pretreated with acid 3% WPB (Table A1, Appendix A).

The TS and VS contents of the samples used in the experiments are presented Table 2. The TS content of untreated banknote wastepaper varied between 915 g/kg (WPB) and 956 g/kg (WPD). For the remaining samples, the amount of dry matter differed from 918 g/kg and 970 g/kg. The amount of VS for untreated WPB was 955 g/kgTS and for untreated WPD 970 g/kgTS. The VS content was the lowest for WPB samples that were pretreated with acid 3% (903 g/kgTS), and highest for WPD samples that were pretreated with NED 200 °C (979 g/kgTS).

### 3.2. Glucose Content from Pretreatment and Ethanol Content after Fermentation

**Figure 2:**
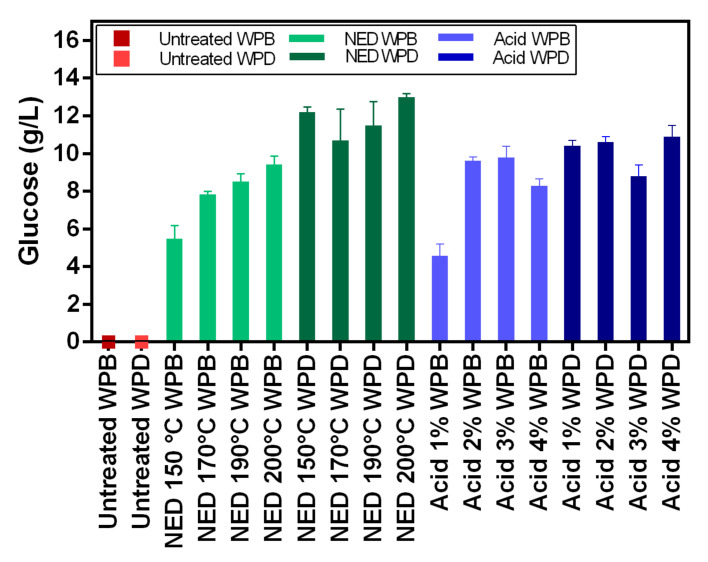
Glucose concentration for hydrolyzed WPB and WPD samples that were pretreated with NED at 150 °C, 170 °C, 190 °C, and 200 °C, and with acid 1%, 2%, 3%, and 4%.

The increase in temperature used for the NED pretreatment of WPB resulted in a more efficient cellulose conversion to glucose as its concentration raised from 5.5 ± 0.71 g/L at 150 °C to 9.4 ± 0.44 g/L at 200 °C (Figure 2). The same trend was not observed for WPD as different temperatures used for the NED pretreatment did not result in statistically different (*p* > 0.05) glucose concentrations (Table A2, Appendix A). The use of NED pretreatment in WPD resulted in the highest glucose concentration of all studies (13 ± 0.19 g/L). The glucose yield of acid pretreated WPB showed a correlation with the acid concentration. The acid concentration of 2 and 3% resulted in 9.6 ± 0.18 and 9.8 ± 0.61 g/L of glucose, respectively. These concentrations were not different statistically and probably represent the range of optimum acid concentration for WPB pretreatment, as the performances of pretreatment using 1 and 4% of acid were inferior. The acid concentration on WPD pretreatment resulted in no significant statistical difference between the glucose concentrations obtained when using 1, 2, and 4% of acid (10.6 g/L of glucose on average). Unexpectedly, the pretreatment with 3% of acid pretreatment in WPD resulted in a lower glucose concentration (8.8 ± 0.60 g/L). 

**Figure 3:**
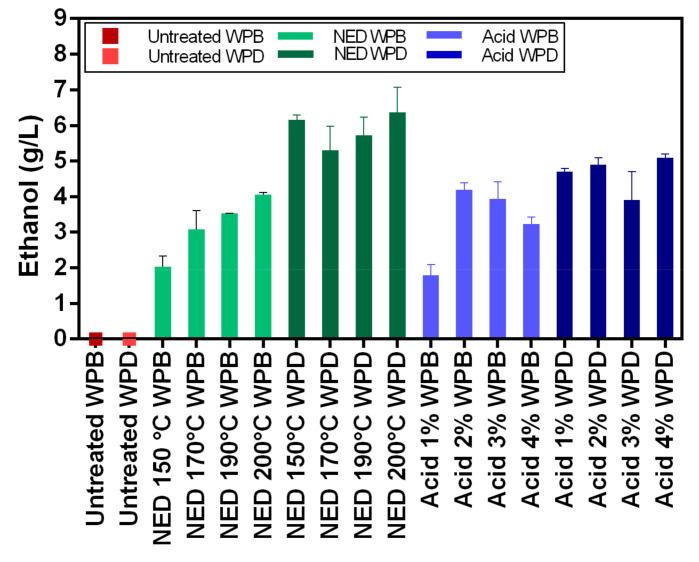
The concentrations of ethanol after the fermentation stage for WPB and WPD samples that were pretreated with NED at 150 °C, 170 °C, 190 °C, and 200 °C and with acid 1%, 2%, 3%, and 4%.

The glucose-containing liquid phases from the different pretreatments of WPB and WPD were used as a substrate for bioethanol production using *S. cerevisiae* (Figure 3). As expected, the ethanol concentration reflected the initial glucose concentration. The highest ethanol concentration was obtained from the fermentation using WPD pretreated with NED (6.4 ± 0.72 g/L), however there was no statistical difference amongst the different temperatures used (Table A3, Appendix A). The ethanol yield from glucose was also the highest, 0.5 g ethanol/g glucose (98% of theoretical yield). The second highest ethanol concentration and yield were also from WPD using acid pretreatment, 4.9 ± 0.20 g/L and 0.46 g ethanol/g glucose (90% of the theoretical yield). The best results with WPB were when using pretreatments with 2% of acid and NED at 200 °C, both about 4 g/L of ethanol and 0.43 g ethanol/g glucose (84% of the theoretical yield). 

When it comes to the hydrolysis efficiency (Table 3), the results show relatively low values (between 5–15%). This can be attributed to the nature of the banknote paper. The cotton structure (main component of banknote paper) is highly crystalline in nature and thus, recalcitrant to enzymatic hydrolysis. Both pretreatments have only limited effect on reducing crystallinity of the cellulose in the paper. The highest hydrolysis efficiencies were obtained with acid pretreatment of WPB samples however, this is due to the fact that in the deinking process some of the amorphous cellulose is lost. On the other hand, the fermentation efficiencies of these same samples are lower due to the presence of inhibitory compounds originating from the ink. Fermentation efficiency varies between 73% and 99% and is clearly higher for deinked samples. 

### 3.3. Potential of Waste Banknote Paper for Biomethane Production

#### 3.3.1. Methane Yields

**Figure 4:**
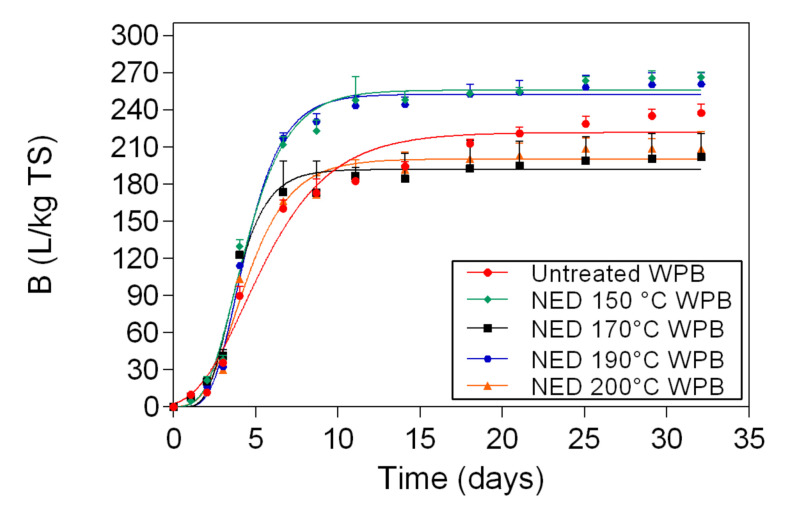
Biomethane potential measurement results and respective fitting curves for untreated samples and for WPB samples that were pretreated with NED at 150 °C, 170 °C, 190 °C, and 200 °C.

In Figure 4 the biomethane potentials for untreated sample and for WPB samples that were pretreated with NED at 150 °C, 170 °C, 190 °C, and 200 °C are presented. Samples that were pretreated at 150 °C had the highest biomethane yields (266 L/kg TS), followed by those that were pretreated at 190 °C (261 L /kg TS), untreated material (238 L/kg TS), 200 °C (208 L/kg TS), and 170 °C (202 L/kg TS).

**Figure 5:**
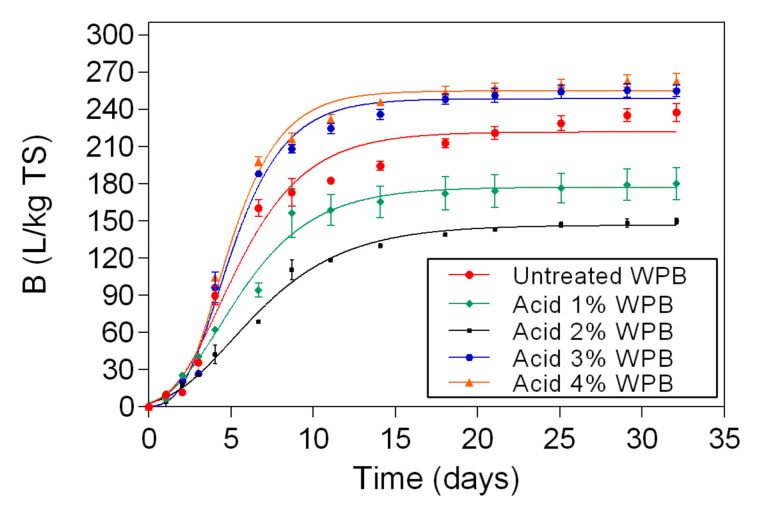
Biomethane potential measurement results and respective fitting curves for untreated samples and for WPB samples that were pretreated with acid at 1%, 2%, 3%, and 4%.

The biomethane results of untreated sample and WPB samples that were pretreated with acid at 1%, 2%, 3%, and 4% are shown in Figure 5. The biomethane yield varied between 150 L/kg TS (samples that were pretreated with acid 2%) and 263 L/kg TS (samples that were pretreated with acid 4%). 

**Figure 6:**
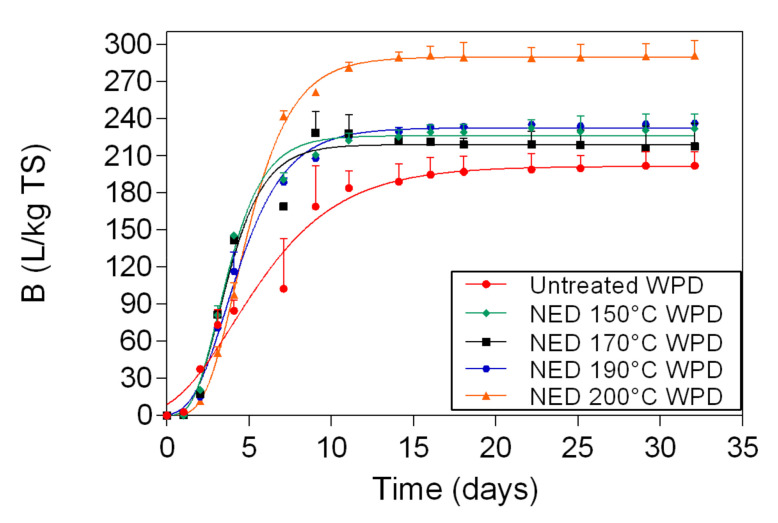
Biomethane potential measurement results and respective fitting curves for untreated samples and for WPD samples that were pretreated with NED at 150 °C, 170 °C, 190 °C, and 200 °C.

The biomethane potential of untreated WPD and of samples that were pretreated with NED (WPD) is illustrated in Figure 6. Untreated WPD had the lowest biomethane yields (202 L/kg TS), while samples that were pretreated with NED at 200 °C (WPD) had the highest biomethane yields (291 L/kg TS). The biomethane yields of samples that were pretreated with NED 170 °C (WPD) was 217 L/kg TS, followed by samples that were pretreated with NED 150 °C (WPD) (232 L/kg TS), and NED 190 °C (WPD) (236 L/kg TS). Statistically significant differences were found between untreated material and samples that were pretreated with NED at different temperatures (Table A4, Appendix A).

**Figure 7:**
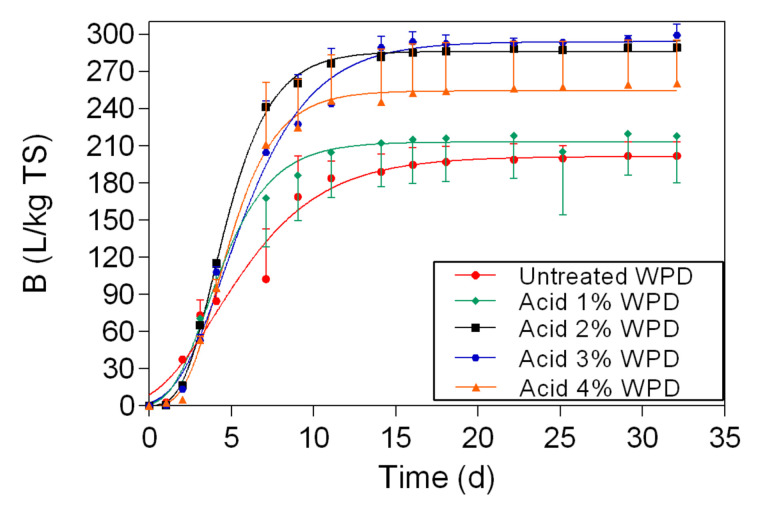
Biomethane potential measurement results and respective fitting curves for untreated samples and for WPD samples that were pretreated with acid at 1%, 2%, 3%, and 4%.

The biomethane potential of untreated WPD and samples that were pretreated with acid at 1%, 2%, 3%, and 4% (WPD) is reported in Figure 7. The biomethane yield of WPD was 202 L/kg TS, followed by samples that were pretreated with 1% acid WPD (218 L/kg TS), 4% acid WPD (260 L/kg TS), 2% acid WPD (289 L/kg TS), and 3% acid WPD (299 L/kg TS). Statistically significant differences were found between untreated WPD and samples pretreated with acid (Table A4, Appendix A).

**Figure 8:**
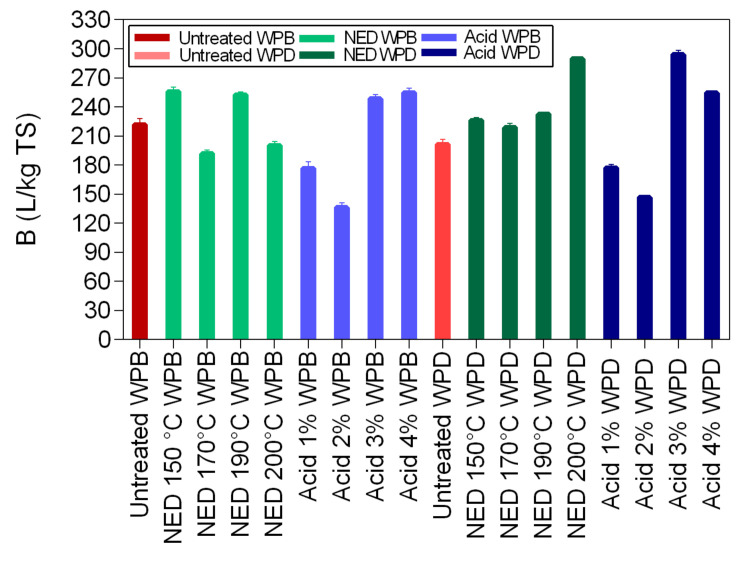
Maximum methane yields (B_max_) of the fitting curves for WPB and WPD samples that were pretreated with NED at 150 °C, 170 °C, 190 °C, and 200 °C and with acid at 1%, 2%, 3%, and 4%.

The maximum methane yield for WPB samples that were pretreated with NED at 150 °C, 170 °C, 190 °C, and 200 °C and with acid at 1%, 2%, 3%, and 4% shown in Figure 8. The maximum methane yield of untreated WPB was 222 L/kg TS. For samples (WPB) that were pretreated with NED, the maximum methane yields varied between 192 L/kg TS (samples that were pretreated with NED at 170 °C) and 256 L/kg TS (samples that were pretreated with NED at 150 °C). For samples (WPB) that were pretreated with acid the maximum methane yield was lower at 2% (136 L/kg TS) and higher at 4% (255 L/kg TS). The maximum methane yield of untreated WPD was 201 L/kg TS. For samples (WPD) that were pretreated with NED, the maximum methane yields ranged between 219 L/kg TS (NED 170 °C WPD) and 290 L/kg TS (NED 200 °C WPD), while for samples (WPD) that were pretreated with acid it ranged between 147 L/kg TS (acid 2% WPD) and 294 L/kg TS (acid 3% WPD). Statistically significant differences were found between NED 200 °C WPD vs. NED 200 °C WPB and Acid 2% WPD vs. Acid 2% WPB (Table A4, Appendix A). 

#### 3.3.2. Digestion Time 

The digestion time to reach 85% (B_85_) and 95% (B_95_) of maximum biomethane potential and the Gompertz growth parameters for WPB and WPD samples that were pretreated with NED at 150 °C, 170 °C, 190 °C, and 200 °C and with acid at 1%, 2%, 3%, and 4% are shown in Table 4. Untreated WPB needed 14 days (201 L/kg TS) to achieve B85 and 21 days (225 L/kg TS) to reach B95, while untreated WPD required 7.4 days (172 L/kg TS) to achieve B85 and 11 days (193 L/kg TS) to reach B95. Overall, WPB samples that were pretreated with 1% acid and WPB samples that were pretreated with NED 150 °C and NED 170 °C require less time to achieve B85. WPD samples that were pretreated with 3% acid and WPB samples that were pretreated with 3% acid and 4% acid need more time to reach B85. A similar trend is followed for B95.

Untreated WPB needed 14 days (201 L/kg TS) to achieve B_85_ and 21 days (225 L/kg TS) to reach B_95_, while untreated WPD required 7.4 days (172 L/kg TS) to achieve B_85_ and 11 days (193 L/kg TS) to reach B_95_. Overall, WPB samples that were pretreated with 1% acid and WPB samples that were pretreated with NED 150 °C and NED 170 °C require less time to achieve B_85_. WPD samples that were pretreated with 3% acid and WPB samples that were pretreated with 3% acid and 4% acid need more time to reach B_85_. A similar trend is followed for B_95_.

## 4. Discussion

### 4.1. Chemical Composition

The cellulose content reported in this study was 6% to 18% lower than the values reported in the literature [314 however, the overall percentage of cellulose was still very high (>77%). The high percentage of cellulose that was found in the waste banknote paper is due to its composition. Waste banknote paper has high amounts of cotton, which has mainly cellulose on its composition. The results obtained in this study were also compared with the chemical composition of cotton crops reported by Rocha-Meneses et al. [15]. The authors described the content of cellulose and hemicellulose present in cotton as 80–95% and 5–20%, respectively, which is in line with the results found in our study. These results are favorable for bioethanol and biogas production since research has shown that high amounts of cellulose contribute to high yields of bioethanol and biomethane. The hemicellulose and lignin contents are within the range of values reported in the literature [31]. Moreover, low lignin content is advantageous since the amount of energy that is required to break down the chemical bonds between hemicellulose and lignin and to make cellulose accessible for the enzymatic hydrolysis is low [20,32].

The amounts of TS and VS are fundamental when it comes to biogas production. High amounts of TS specify the amount of substrate that is accessible for the anaerobic digestion process, while high amounts of VS refer to the amount of substrate that can be transformed into biomethane. When compared with similar subtracts, higher amounts of VS lead to higher amounts of biogas [32,33]. 

### 4.2. Glucose Content after Hydrolysis and Ethanol Content after Fermentation

The use of WPD resulted in higher glucose and ethanol concentrations and yields when compared to WPB regardless of the pretreatment. A previous study also showed that the deinking process improved ethanol production of the pretreated waste banknote [26]. In this present work, when fermenting the NED-pretreated WPD, we were able to achieve 6.36 g/L of ethanol and 98% of the theoretical ethanol yield from glucose (0.50 g ethanol/g glucose). Although the ethanol concentration achieved is lower than the one obtained by Rocha-Meneses et al. [32] when using NED pretreatment to Napier grass as a fermentation substrate (10.3 g/L), a lower bioethanol yield (90% of the theoretical yield) was reported by the authors, probably by the presence of microbial growth inhibitors. No microbial inhibition effect was observed when using pretreated WPD as a fermentation substrate. Jeihanipour and Taherzadeha (2009) [34] reported a yield of 0.48 g ethanol/g textile from alkali pretreated cotton linter and waste jeans as a substrate for *S. cerevisiae* during a simultaneous saccharification and fermentation process (SSF). The yield reported by Jeihanipour and Taherzadeha (2009) [34] is higher than the one obtained in the present work (0.21 g ethanol/g waste banknote), but this yield can possibly be enhanced by optimizing the enzymatic hydrolysis step increasing glucose content, and, therefore, the ethanol production, as shown previously by Aghmashhadi et al. (2020) [26], widening the potential of this waste biomass utilization.

### 4.3. Potential of Waste Banknote Paper for Biomethane Production

From Figure 4 it is evident that samples of WPB that were pretreated with NED at 150 °C and 190 °C produce 12–13% more biomethane than untreated WPB, while samples that were pretreated at 170 °C and 200 °C have biomethane yields 9–15% lower than untreated WPB. At the moment, the mechanism behind these differences is not clear. As there is clearer trend in WPD, the hectic results of WPB might be due to the presence of ink and its interactions with the other compounds that are produced during the pretreatment at high temperature. Moreover, it might be due to the different amounts of ink present in different samples of WPB. Not all of the banknote is uniformly covered with the same kind of ink. Furthermore, these pretreatment methods are effective only under certain temperatures, which could require further studies in order to investigate the optimum pretreatment temperature for the anaerobic digestion process [35,36]. If the experiments are performed outside the optimum pretreatment temperatures it can lead to the production of inhibitory compounds and a decrease in the substrate biodegradability [37,38]. 

As it can be seen from Figure 5 only pretreatment with 3% and 4% acid increased biomethane production compared to untreated sample for WPB. Samples that were pretreated with high acid concentrations showed biomethane content 10–12% higher than untreated banknote paper. Similar results were reported by Sarto, Hildayati and Syaichurrozi (2019) [39]. On the other hand, lower concentrations of acid in the pretreatment seems to inhibit the biomethane production. This can be due to the composition of the samples. As the samples still have ink in their composition, probably harsher pretreatment methods are required to disrupt the structure of the banknote paper and access the cellulose, as well as longer retention times, since the degradation rate will be slower. Moreover, the utilization of acid to adjust the pH can have led to excessive sugar degradation and the production of inhibitory compounds [40]. A study reported by Venturin et al. [41] showed that H_2_SO_4_ pretreatment inhibited biogas production. In order to ascertain the exact mechanism, further studies should be performed, with different retention times in the acid pretreatment, and different reagents of pH adjustment.

The biomethane yields of samples that were pretreated with NED (WPD) (Figure 6) were 8-44% higher than the biomethane yields of untreated material. As it is shown in Figure 7, the biomethane content of samples that where pretreated with acid (WPD) was 8-48% higher than the biomethane content of untreated banknote paper. Overall, the biomethane yields of deinking samples (NED WPD and acid WPD) were highly improved, when compared with blank samples (NED WPB and acid WPB). For samples that were pretreated with NED, the biomethane yields of WPD were 8–35% higher than the biomethane yields of WPB. For samples that were pretreated with acid, the biomethane yields of WPD were 17–93% higher than the biomethane yields of WPB. These differences between WPB and WPD samples can be explained by the deinking process. The ink removal uses alkali as a solution. This means that deinking samples are subjected to two combined pretreatment methods alkali and NED. This can leave waste banknote paper with an increased amorphous structure, higher traits and quality, which will increase its biodegradability by the anaerobic microorganisms and as a result, increase biomethane yields.

As it is evident from Figure 8, samples that were pretreated with acid 2% WPB, acid 1% WPB, NED 170 °C WPB, NED 200 °C WPB, had the lowest biomethane yields (between 136 L/kg TS and 200 L/kg TS), while samples that were pretreated with acid 4% WPD, NED 200 °C WPD, and acid 3% WPD had the highest biomethane yields (between 254 mol/kg TS and 294 mol/kg TS). These results can be explained by the deinking process, by the effect of high acid concentrations, and by the effect of high pretreatment temperatures. Research has shown that the presence on ink in waste paper inhibits the enzymatic hydrolysis, and reduces the amount of sugars available for the fermentation stage, which results in inefficient bioethanol production [42]. Therefore, as expected, samples without ink resulted in better yields than samples with ink. On the other hand, high acid concentrations reduce the crystalline structure of cellulose more efficiently, making it more accessible for the enzymatic hydrolysis, which will lead to higher bioethanol and biogas yields [39,43,44]. The temperature also plays a role in biomethane yields. Research has shown that cellulose solubilization increases proportionally with the increment of the pretreatment temperature [39,45]. 

The differences in the digestion time between WPD and WPB can be explained by the presence of ink. Research has shown that the presence of ink can inhibit biorefinery processes [26].

These results are particularly important when it comes to the reduction of environmental impacts caused by the incineration or landfilling of waste banknote paper. At the moment, these are the most common handling options that are being applied to waste banknote paper. However, incineration and landfilling are a source of environmental pollution, contaminating the air, soil and water. Therefore, the Central Bank of Iran has decided to take action and reduce its environmental footprint caused by waste banknote paper and shift to waste management solutions that are more environmentally friendly. Therefore, biogas and bioethanol production are proposed as alternative handling options for these residues since it is known that they have a lower footprint when compared to incineration or landfilling [46]. Further research should be performed in order to make this solution competitive and to quantify the energy output from biogas, bioethanol, incineration and landfilling of waste banknote paper.

## 5. Conclusions

In this study, two different pretreatment methods (chemical and physio-chemical) were used to investigate the potential of waste banknote paper with and without ink for bioethanol and biomethane production. The results of this study show that glucose and ethanol concentrations are higher in WPD samples than for WPB. The glucose yields varied between 4.57 g/L (1% acid WPB) and 12.98 g/L (NED 200 °C WPD), while ethanol yields varied between 1.8 g/L (1% Acid WPB) and 6.36 g/L (NED 200 °C WPD). For WPB samples, the highest biomethane yields were reported in samples that were pretreated with NED 150 °C (266 L/kg TS) and 4% acid (263 L/Kg TS). For WPD samples, samples that were pretreated with 3% acid (299 L/Kg TS) and NED 200 °C (292 L/Kg TS) pretreatments insured the highest biomethane production. In general, the biomethane potential of WPD samples was higher than that of similarly pretreated WPB samples. NED pretreatment method gives higher glucose, ethanol, and biomethane yields than samples that were pretreated with sulfuric acid. The deinking process, the acid concentration, and the pretreatment temperature all influence glucose, bioethanol, and biomethane yields. More research should be done to find the most efficient ways to increase glucose, bioethanol, and biomethane yields. 

## Figures and Tables

**Table 1:** Hemicellulose, cellulose, lignin, ash, and moisture content of the samples after the pretreatment stage.

Description	Cellulose (%)	Hemicellulose (%)	Lignin (%)	pH	Weight Loss (%)
NED 150 °C WPB	85 ± 3	6 ± 3	1 ± 0.7	5.5 ± 0.3	5.2
NED 170 °C WPB	84 ± 0.2	4 ± 2	1 ± 0.6	5.5 ± 0.2	6.1
NED 190 °C WPB	78 ± 5	4 ± 0.5	0.9 ± 0.0	5.7 ± 0.0	6.7
NED 200 °C WPB	83 ± 1	3 ± 0.5	0.6 ± 0.0	5.5 ± 0.0	6.3
Acid 1% WPB	85 ± 1	3 ± 2	0.7 ± 0.0	5.9 ± 0.1	6.2
Acid 2% WPB	83 ± 0.6	1 ± 3	0.6 ± 0.0	5.7 ± 0.0	9.4
Acid 3% WPB	70 ± 7	1 ± 1	0.7 ± 0.0	6.0 ± 0.0	10.2
Acid 4% WPB	79 ± 4	2 ± 2	0.6 ± 0.2	5.8 ± 0.0	9.7
NED 150 °C WPD	89 ± 0.6	8 ± 0.0	0.2 ± 0.1	5.6 ± 0.0	0.6
NED 170 °C WPD	88 ± 0.1	2± 1	0.3 ± 0.1	5.5 ± 0.3	3.1
NED 190 °C WPD	89 ± 2	2 ± 1.6	0.0 ± 0.0	5.5 ± 0.3	5.0
NED 200 °C WPD	87± 12	1 ± 0.3	0.0 ± 0.0	6.0 ± 0.0	9.3
Acid 1% WPD	88 ± 0.8	3 ± 1	0.5 ± 0.1	5.6 ± 0.4	2.4
Acid 2% WPD	88 ± 0.0	3 ± 0.4	0.3 ± 0.0	5.3 ± 0.1	1.7
Acid 3% WPD	84 ± 2	2 ± 1	0.2 ± 0.1	5.8 ± 0.1	2.1
Acid 4% WPD	86 ± 0.0	4 ± 0.0	0.2 ± 0.0	5.7 ± 0.4	2.6
Untreated WPB	77 ± 2	7 ± 3	2 ± 0.2	5.5 ± 0.5	-
Untreated WPD	89 ± 0.4	3 ± 0.4	1 ± 0.1	5.6 ± 0.2	-

**Table 3:** Hydrolysis and fermentation efficiency.

	Hydrolysis Efficiency	Fermentation Efficiency
NED 150 °C WPB	6	73
NED 170 °C WPB	8	77
NED 190 °C WPB	10	81
NED 200 °C WPB	10	84
Acid 1% WPB	13	77
Acid 2% WPB	12	85
Acid 3% WPB	15	79
Acid 4% WPB	15	76
NED 150 °C WPD	5	99
NED 170 °C WPD	10	97
NED 190 °C WPD	10	98
NED 200 °C WPD	9	96
Acid 1% WPD	11	89
Acid 2% WPD	11	91
Acid 3% WPD	9	87
Acid 4% WPD	11	92

**Table 4:** Digestion time to achieve 85% (B_85_) and 95% (B_95_) of maximum biomethane potential and Gompertz growth parameters for WPB and WPD that were pretreated with NED at 150 °C, 170 °C, 190 °C, and 200 °C and with acid at 1%, 2%, 3%, and 4%.

	B_85_		B_95_		Gompertz Growth		
	L/kg TS	Days	L/kg TS	Days	Y_M_	Y_0_	K
NED 150 °C WPB	231	11	258	17	256 ± 4	0.06 ± 0.1	0.6 ± 0.07
NED 170 °C WPB	173	10	193	16	192 ± 4	0.0002 ± 0.001	0.8 ± 0.1
NED 190 °C WPB	228	11	255	17	253 ± 3	0.001 ± 0.003	0.6 ± 0.06
NED 200 °C WPB	181	12	203	18	200 ± 4	0.09 ± 0.2	0.5 ± 0.08
Acid 1% WPB	154	7.7	172	12	177 ± 3	3 ± 2	0.3 ± 0.03
Acid 2% WPB	126	11	140	17	147 ± 3	3 ± 1	0.3 ± 0.02
Acid 3% WPB	224	13	251	19	249 ± 4	0.3 ±0.4	0.4 ± 0.04
Acid 4% WPB	230	13	257	19	255 ± 4	0.2 ± 0.3	0.5 ± 0.05
NED 150 °C WPD	202	9.7	226	15	226 ± 3	0.08 ± 0.1	0.7 ± 0.07
NED 170 °C WPD	195	8.0	218	12	219 ± 4	0.08 ± 0.2	0.6 ± 0.1
NED 190 °C WPD	207	11	232	17	232 ± 2	0.6 ± 0.5	0.5 ± 0.04
NED 200 °C WPD	260	12	291	18	290 ± 1	0.02 ± 0.01	0.5 ± 0.02
Acid 1% WPD	190	11	212	17	213 ± 3	1 ± 1	0.5 ± 0.05
Acid 2% WPD	256	12	286	17	286 ± 1	0.1 ± 0.06	0.5 ± 0.02
Acid 3% WPD	264	14	295	21	294 ± 4	2 ± 1	0.3 ± 0.03
Acid 4% WPD	229	12	256	18	254 ± 2	0.07 ± 0.07	0.5 ± 0.03
Untreated WPB	201	14	225	21	222 ± 6	3 ± 2	0.3 ± 0
Untreated WPD	172	7.4	193	11	201 ± 5	9 ± 4	0.3 ± 0.04

**Table 2:** Total Solids and volatile solids content.

Substrate	TS (g/kg)	VS (g/KgTS)
NED 150 °C WPB	960 ± 5	963 ± 9
NED 170 °C WPB	957 ± 9	944 ± 13
NED 190 °C WPB	934 ± 11	945 ± 1
NED 200 °C WPB	958 ± 6	940 ± 3
Acid 1% WPB	947 ± 8	956 ± 7
Acid 2% WPB	934 ± 4	930 ± 3
Acid 3% WPB	929 ± 7	903 ± 6
Acid 4% WPB	970 ± 1	917 ± 8
NED 150 °C WPD	959 ± 14	966 ± 2
NED 170 °C WPD	954 ± 0	971 ± 2
NED 190 °C WPD	962 ± 6	973 ± 0
NED 200 °C WPD	961 ± 0	979 ± 0
Acid 1% WPD	953 ± 6	972 ± 3
Acid 2% WPD	945 ± 2	950 ± 1
Acid 3% WPD	918 ± 5	937 ± 0
Acid 4% WPD	952 ± 1	916 ± 0
Untreated WPB	915 ± 7	955 ± 16
Untreated WPD	953 ± 3	970 ± 0

## Data Availability

Not applicable.

## Appendix A

**Table A1:** Two-way ANOVA, Tukey’s multiple comparisons test, for cellulose content.

Dunn’s Multiple Comparisons Test	Mean Rank Diff.	Significant?	Summary	Adjusted *p* Value
Acid 3% WPB vs. NED 150 °C WPB	−29.58	Yes	****	<0.0001
Acid 3% WPB vs. NED 170 °C WPB	−28.6	Yes	***	0.0001
Acid 3% WPB vs. NED 190 °C WPB	−22.89	Yes	**	0.0074
Acid 3% WPB vs. NED 200 °C WPB	−27.8	Yes	***	0.0002
Acid 3% WPB vs. NED 150 °C WPD	−33.52	Yes	****	<0.0001
Acid 3% WPB vs. NED 170 °C WPD	−32.84	Yes	****	<0.0001
Acid 3% WPB vs. NED 190 °C WPD	−32.67	Yes	****	<0.0001
Acid 3% WPB vs. NED 200 °C WPD	−32.33	Yes	****	<0.0001
Acid 3% WPB vs. Acid 1% WPB	−29.52	Yes	****	<0.0001
Acid 3% WPB vs. Acid 2% WPB	−27.38	Yes	***	0.0003
Acid 4% WPB vs. Acid 3% WPB	23.46	Yes	**	0.0052
Acid 1% WPD vs. Acid 3% WPB	33.26	Yes	****	<0.0001
Acid 2% WPD vs. Acid 3% WPB	32.5	Yes	****	<0.0001
Acid 3% WPD vs. Acid 3% WPB	29.23	Yes	****	<0.0001
Acid 4% WPD vs. Acid 3% WPB	30.37	Yes	****	<0.0001
Untreated WPB vs. Acid 3% WPB	22.15	Yes	*	0.0118
Untreated WPD vs. Acid 3% WPB	33.76	Yes	****	<0.0001

* *p* ≤ 0.05, ** *p* ≤ 0.01, *** *p* ≤ 0.001, **** *p* ≤ 0.0001.

**Table A2:** Two-way ANOVA, Tukey’s multiple comparisons test, for glucose yields.

Dunn’s Multiple Comparisons Test	Mean Rank Diff.	Significant?	Summary	Adjusted *p* Value
NED 150 °C WPB vs. NED 170 °C WPB	−2.36	Yes	***	0.0002
NED 150 °C WPB vs. NED 190 °C WPB	−3.02	Yes	****	<0.0001
NED 150 °C WPB vs. NED 200 °C WPB	−3.95	Yes	****	<0.0001
NED 150 °C WPB vs. NED 150 °C WPD	−6.72	Yes	****	<0.0001
NED 150 °C WPB vs. NED 170 °C WPD	−5.21	Yes	****	<0.0001
NED 150 °C WPB vs. NED 190 °C WPD	−6.02	Yes	****	<0.0001
NED 150 °C WPB vs. NED 200 °C WPD	−7.5	Yes	****	<0.0001
NED 150 °C WPB vs. Acid 2% WPB	−4.15	Yes	****	<0.0001
NED 150 °C WPB vs. Acid 3% WPB	−4.3	Yes	****	<0.0001
NED 150 °C WPB vs. Acid 4% WPB	−2.8	Yes	****	<0.0001
NED 150 °C WPB vs. Acid 1% WPD	−4.92	Yes	****	<0.0001
NED 150 °C WPB vs. Acid 2% WPD	−5.12	Yes	****	<0.0001
NED 150 °C WPB vs. Acid 3% WPD	−3.32	Yes	****	<0.0001
NED 150 °C WPB vs. Acid 4% WPD	−5.42	Yes	****	<0.0001
NED 170 °C WPB vs. NED 150 °C WPD	−4.36	Yes	****	<0.0001
NED 170 °C WPB vs. NED 170 °C WPD	−2.85	Yes	****	<0.0001
NED 170 °C WPB vs. NED 190 °C WPD	−3.66	Yes	****	<0.0001
NED 170 °C WPB vs. NED 200 °C WPD	−5.14	Yes	****	<0.0001
NED 170 °C WPB vs. Acid 1% WPB	3.27	Yes	****	<0.0001
NED 170 °C WPB vs. Acid 2% WPB	−1.79	Yes	*	0.0164
NED 170 °C WPB vs. Acid 3% WPB	−1.94	Yes	**	0.0058
NED 170 °C WPB vs. Acid 1% WPD	−2.56	Yes	****	<0.0001
NED 170 °C WPB vs. Acid 2% WPD	−2.76	Yes	****	<0.0001
NED 170 °C WPB vs. Acid 4% WPD	−3.06	Yes	****	<0.0001
NED 190 °C WPB vs. NED 150 °C WPD	−3.7	Yes	****	<0.0001
NED 190 °C WPB vs. NED 170 °C WPD	−2.19	Yes	***	0.0009
NED 190 °C WPB vs. NED 190 °C WPD	−3	Yes	****	<0.0001
NED 190 °C WPB vs. NED 200 °C WPD	−4.48	Yes	****	<0.0001
NED 190 °C WPB vs. Acid 1% WPB	3.93	Yes	****	<0.0001
NED 190 °C WPB vs. Acid 1% WPD	−1.9	Yes	**	0.0077
NED 190 °C WPB vs. Acid 2% WPD	−2.1	Yes	**	0.0018
NED 190 °C WPB vs. Acid 4% WPD	−2.4	Yes	***	0.0002
NED 200 °C WPB vs. NED 150 °C WPD	−2.77	Yes	****	<0.0001
NED 200 °C WPB vs. NED 190 °C WPD	−2.07	Yes	**	0.0022
NED 200 °C WPB vs. NED 200 °C WPD	−3.55	Yes	****	<0.0001
NED 200 °C WPB vs. Acid 1% WPB	4.86	Yes	****	<0.0001
NED 150 °C WPD vs. Acid 1% WPB	7.63	Yes	****	<0.0001
NED 150 °C WPD vs. Acid 2% WPB	2.57	Yes	****	<0.0001
NED 150 °C WPD vs. Acid 3% WPB	2.42	Yes	***	0.0001
NED 150 °C WPD vs. Acid 4% WPB	3.92	Yes	****	<0.0001
NED 150 °C WPD vs. Acid 1% WPD	1.8	Yes	*	0.0154
NED 150 °C WPD vs. Acid 3% WPD	3.4	Yes	****	<0.0001
NED 170 °C WPD vs. NED 200 °C WPD	−2.29	Yes	***	0.0004
NED 170 °C WPD vs. Acid 1% WPB	6.12	Yes	****	<0.0001
NED 170 °C WPD vs. Acid 4% WPB	2.41	Yes	***	0.0002
NED 170 °C WPD vs. Acid 3% WPD	1.89	Yes	**	0.0083
NED 190 °C WPD vs. Acid 1% WPB	6.93	Yes	****	<0.0001
NED 190 °C WPD vs. Acid 2% WPB	1.87	Yes	**	0.0095
NED 190 °C WPD vs. Acid 3% WPB	1.72	Yes	*	0.026
NED 190 °C WPD vs. Acid 4% WPB	3.22	Yes	****	<0.0001
NED 190 ° C WPD vs. Acid 3% WPD	2.7	Yes	****	<0.0001
NED 200 °C WPD vs. Acid 1% WPB	8.41	Yes	****	<0.0001
NED 200 °C WPD vs. Acid 2% WPB	3.35	Yes	****	<0.0001
NED 200 °C WPD vs. Acid 3% WPB	3.2	Yes	****	<0.0001
NED 200 °C WPD vs. Acid 4% WPB	4.7	Yes	****	<0.0001
NED 200 °C WPD vs. Acid 1% WPD	2.58	Yes	****	<0.0001
NED 200 °C WPD vs. Acid 2% WPD	2.38	Yes	***	0.0002
NED 200 °C WPD vs. Acid 3% WPD	4.18	Yes	****	<0.0001
NED 200 °C WPD vs. Acid 4% WPD	2.08	Yes	**	0.0021
Acid 1% WPB vs. Acid 2% WPB	−5.06	Yes	****	<0.0001
Acid 1% WPB vs. Acid 3% WPB	−5.21	Yes	****	<0.0001
Acid 1% WPB vs. Acid 4% WPB	−3.71	Yes	****	<0.0001
Acid 1% WPB vs. Acid 1% WPD	−5.83	Yes	****	<0.0001
Acid 1% WPB vs. Acid 2% WPD	−6.03	Yes	****	<0.0001
Acid 1% WPB vs. Acid 3% WPD	−4.23	Yes	****	<0.0001
Acid 1% WPB vs. Acid 4% WPD	−6.33	Yes	****	<0.0001
Acid 4% WPB vs. Acid 1% WPD	−2.12	Yes	**	0.0015
Acid 4% WPB vs. Acid 2% WPD	−2.32	Yes	***	0.0003
Acid 4% WPB vs. Acid 4% WPD	−2.62	Yes	****	<0.0001
Acid 2% WPD vs. Acid 3% WPD	1.8	Yes	*	0.0154
Acid 3% WPD vs. Acid 4% WPD	−2.1	Yes	**	0.0018

* *p* ≤ 0.05, ** *p* ≤ 0.01, *** *p* ≤ 0.001, **** *p* ≤ 0.0001.

**Table A3:** Two-way ANOVA, Tukey’s multiple comparisons test, for ethanol yields.

Dunn’s Multiple Comparisons Test	Mean Rank Diff.	Significant?	Summary	Adjusted *p* Value
NED 150 °C WPB vs. NED 200 °C WPB	−2.02	Yes	**	0.0032
NED 150 °C WPB vs. NED 150 °C WPD	−4.12	Yes	****	<0.0001
NED 150 °C WPB vs. NED 170 °C WPD	−3.28	Yes	****	<0.0001
NED 150 °C WPB vs. NED 190 °C WPD	−3.69	Yes	****	<0.0001
NED 150 °C WPB vs. NED 200 °C WPD	−4.33	Yes	****	<0.0001
NED 150 °C WPB vs. Acid 2% WPB	−2.16	Yes	**	0.0011
NED 150 °C WPB vs. Acid 3% WPB	−1.9	Yes	**	0.0077
NED 150 °C WPB vs. Acid 1% WPD	−2.67	Yes	****	<0.0001
NED 150 °C WPB vs. Acid 2% WPD	−2.87	Yes	****	<0.0001
NED 150 °C WPB vs. Acid 3% WPD	−1.87	Yes	**	0.0095
NED 150 °C WPB vs. Acid 4% WPD	−3.07	Yes	****	<0.0001
NED 170 °C WPB vs. NED 150 °C WPD	−3.06	Yes	****	<0.0001
NED 170 °C WPB vs. NED 170 °C WPD	−2.22	Yes	***	0.0007
NED 170 °C WPB vs. NED 190 °C WPD	−2.63	Yes	****	<0.0001
NED 170 °C WPB vs. NED 200 °C WPD	−3.27	Yes	****	<0.0001
NED 170 °C WPB vs. Acid 2% WPD	−1.81	Yes	*	0.0143
NED 170 °C WPB vs. Acid 4% WPD	−2.01	Yes	**	0.0035
NED 190 °C WPB vs. NED 150 °C WPD	−2.62	Yes	****	<0.0001
NED 190 °C WPB vs. NED 170 °C WPD	−1.78	Yes	*	0.0176
NED 190 °C WPB vs. NED 190 °C WPD	−2.19	Yes	***	0.0009
NED 190 °C WPB vs. NED 200 °C WPD	−2.83	Yes	****	<0.0001
NED 190 °C WPB vs. Acid 1% WPB	1.73	Yes	*	0.0244
NED 200 °C WPB vs. NED 150 °C WPD	−2.1	Yes	**	0.0018
NED 200 °C WPB vs. NED 190 °C WPD	−1.67	Yes	*	0.0357
NED 200 °C WPB vs. NED 200 °C WPD	−2.31	Yes	***	0.0003
NED 200 °C WPB vs. Acid 1% WPB	2.25	Yes	***	0.0006
NED 150 °C WPD vs. Acid 1% WPB	4.35	Yes	****	<0.0001
NED 150 °C WPD vs. Acid 2% WPB	1.96	Yes	**	0.005
NED 150 °C WPD vs. Acid 3% WPB	2.22	Yes	***	0.0007
NED 150 °C WPD vs. Acid 4% WPB	2.92	Yes	****	<0.0001
NED 150 °C WPD vs. Acid 3% WPD	2.25	Yes	***	0.0006
NED 170 °C WPD vs. Acid 1% WPB	3.51	Yes	****	<0.0001
NED 170 °C WPD vs. Acid 4% WPB	2.08	Yes	**	0.0021
NED 190 °C WPD vs. Acid 1% WPB	3.92	Yes	****	<0.0001
NED 190 °C WPD vs. Acid 3% WPB	1.79	Yes	*	0.0164
NED 190 °C WPD vs. Acid 4% WPB	2.49	Yes	****	<0.0001
NED 190 °C WPD vs. Acid 3% WPD	1.82	Yes	*	0.0134
NED 200 °C WPD vs. Acid 1% WPB	4.56	Yes	****	<0.0001
NED 200 °C WPD vs. Acid 2% WPB	2.17	Yes	**	0.001
NED 200 °C WPD vs. Acid 3% WPB	2.43	Yes	***	0.0001
NED 200 °C WPD vs. Acid 4% WPB	3.13	Yes	****	<0.0001
NED 200 °C WPD vs. Acid 1% WPD	1.66	Yes	*	0.038
NED 200 °C WPD vs. Acid 3% WPD	2.46	Yes	***	0.0001
Acid 1% WPB vs. Acid 2% WPB	−2.39	Yes	***	0.0002
Acid 1% WPB vs. Acid 3% WPB	−2.13	Yes	**	0.0014
Acid 1% WPB vs. Acid 1% WPD	−2.9	Yes	****	<0.0001
Acid 1% WPB vs. Acid 2% WPD	−3.1	Yes	****	<0.0001
Acid 1% WPB vs. Acid 3% WPD	−2.1	Yes	**	0.0018
Acid 1% WPB vs. Acid 4% WPD	−3.3	Yes	****	<0.0001
Acid 4% WPB vs. Acid 2% WPD	−1.67	Yes	*	0.0357
Acid 4% WPB vs. Acid 4% WPD	−1.87	Yes	**	0.0095

* *p* ≤ 0.05, ** *p* ≤ 0.01, *** *p* ≤ 0.001, **** *p* ≤ 0.0001.

**Table A4:** One-way ANOVA, Dunn’s multiple comparisons test, for biomethane results.

Dunn’s Multiple Comparisons Test	Mean Rank Diff.	Significant?	Summary	Adjusted *p* Value
Untreated WPD vs. NED 200 °C WPD	−225.6	Yes	****	<0.0001
Untreated WPD vs. Acid 2% WPD	−235.7	Yes	****	<0.0001
Untreated WPD vs. Acid 3% WPD	−223.3	Yes	****	<0.0001
Untreated WPD vs. NED 150 °C WPB	−176.6	Yes	**	0.006
Untreated WPD vs. NED 190 °C WPB	−164.1	Yes	*	0.0204
Untreated WPD vs. Acid 4% WPB	−159.7	Yes	*	0.0307
NED 150 °C WPD vs. Acid 2% WPB	195.6	Yes	***	0.0008
NED 170 °C WPD vs. Acid 2% WPB	186.5	Yes	**	0.0022
NED 190 °C WPD vs. Acid 1% WPB	167.2	Yes	*	0.0152
NED 190 °C WPD vs. Acid 2% WPB	223.6	Yes	****	<0.0001
NED 200 °C WPD vs. Acid 1% WPD	182.5	Yes	**	0.0033
NED 200 °C WPD vs. Untreated WPB	173	Yes	**	0.0087
NED 200 °C WPD vs. NED 170 °C WPB	233.3	Yes	****	<0.0001
NED 200 °C WPD vs. NED 200 °C WPB	211.9	Yes	***	0.0001
NED 200 °C WPD vs. Acid 1% WPB	273.6	Yes	****	<0.0001
NED 200 °C WPD vs. Acid 2% WPB	330.1	Yes	****	<0.0001
Acid 1% WPD vs. Acid 2% WPD	−192.6	Yes	**	0.0013
Acid 1% WPD vs. Acid 3% WPD	−180.2	Yes	**	0.0048
Acid 2% WPD vs. Untreated WPB	183.1	Yes	**	0.0036
Acid 2% WPD vs. NED 170 °C WPB	243.4	Yes	****	<0.0001
Acid 2% WPD vs. NED 200 °C WPB	222	Yes	****	<0.0001
Acid 2% WPD vs. Acid 1% WPB	283.7	Yes	****	<0.0001
Acid 2% WPD vs. Acid 2% WPB	340.2	Yes	****	<0.0001
Acid 3% WPD vs. Untreated WPB	170.7	Yes	*	0.0122
Acid 3% WPD vs. NED 170 °C WPB	231	Yes	****	<0.0001
Acid 3% WPD vs. NED 200 °C WPB	209.6	Yes	***	0.0002
Acid 3% WPD vs. Acid 1% WPB	271.3	Yes	****	<0.0001
Acid 3% WPD vs. Acid 2% WPB	327.8	Yes	****	<0.0001
Acid 4% WPD vs. NED 170 °C WPB	156.9	Yes	*	0.0439
Acid 4% WPD vs. Acid 1% WPB	197.2	Yes	***	0.0008
Acid 4% WPD vs. Acid 2% WPB	253.7	Yes	****	<0.0001
Untreated WPB vs. Acid 2% WPB	157.1	Yes	*	0.0432
NED 150 °C WPB vs. NED 170 °C WPB	184.3	Yes	**	0.0031
NED 150 °C WPB vs. NED 200 °C WPB	162.9	Yes	*	0.0255
NED 150 °C WPB vs. Acid 1% WPB	224.6	Yes	****	<0.0001
NED 150 °C WPB vs. Acid 2% WPB	281.1	Yes	****	<0.0001
NED 170 °C WPB vs. NED 190 °C WPB	−171.8	Yes	*	0.011
NED 170 °C WPB vs. Acid 4% WPB	−167.4	Yes	*	0.0168
NED 190 °C WPB vs. Acid 1% WPB	212.1	Yes	***	0.0001
NED 190 °C WPB vs. Acid 2% WPB	268.6	Yes	****	<0.0001
Acid 1% WPB vs. Acid 3% WPB	−181.5	Yes	**	0.0042
Acid 1% WPB vs. Acid 4% WPB	−207.7	Yes	***	0.0002
Acid 2% WPB vs. Acid 3% WPB	−237.9	Yes	****	<0.0001
Acid 2% WPB vs. Acid 4% WPB	−264.1	Yes	****	<0.0001

* *p* ≤ 0.05, ** *p* ≤ 0.01, *** *p* ≤ 0.001, **** *p* ≤ 0.0001.
